# *tert*-Butyl 6-amino-5-cyano-2-(2-meth­oxy­eth­yl)nicotinate

**DOI:** 10.1107/S1600536812014328

**Published:** 2012-04-13

**Authors:** Yi-Ning Chen, Xing-Dong Zhao, Jie Deng, Qin-Geng Li

**Affiliations:** a Laboratory of Pharmaceutical Chemistry, School of Pharmacy, Chongqing Medical University, Chongqing 400016, People’s Republic of China; b Fochon Pharma Inc., 1933 Davis Street, Suite 207, San Leandro, CA 94577, USA; c Chongqing Pharmaceutical Research Institute Co. Ltd, Chongqing 400016, People’s Republic of China

## Abstract

The title compound, C_14_H_19_N_3_O_3_, was synthesized by the reaction of 3-meth­oxy­propionitrile, *tert*-butyl bromo­acetate and eth­oxy­methyl­enemalononitrile. In the crystal, N—H⋯O hydrogen bonds link the mol­ecules into chains propagating along the *b* axis.

## Related literature

For a related structure, see: Wang *et al.* (2007 ▶). For applications of pyridines, see: Spurr (1995 ▶). For background to the synthesis of highly substituted pyridines, see: Chun *et al.* (2009 ▶, 2011 ▶). 
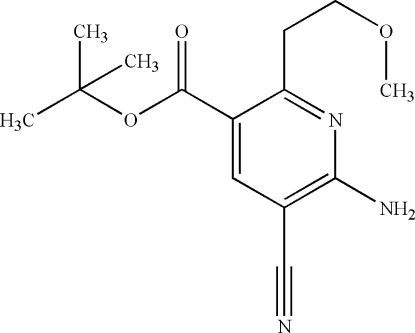


## Experimental

### 

#### Crystal data

C_14_H_19_N_3_O_3_
*M* *_r_* = 277.32
Monoclinic, 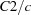

*a* = 10.1155 (4) Å
*b* = 15.2482 (5) Å
*c* = 19.4882 (6) Å
β = 99.853 (3)°
*V* = 2961.59 (18) Å^3^
*Z* = 8
Mo *K*α radiation
μ = 0.09 mm^−1^
*T* = 130 K
0.35 × 0.30 × 0.30 mm

#### Data collection

Agilent Xcalibur Eos diffractometer
Absorption correction: multi-scan (*CrysAlis PRO*; Agilent, 2010 ▶) *T*_min_ = 0.902, *T*_max_ = 1.000
5419 measured reflections
2608 independent reflections
2094 reflections with *I* > 2σ(*I*)
*R*_int_ = 0.020

#### Refinement

*R*[*F* ^2^ > 2σ(*F* ^2^)] = 0.040
*wR*(*F* ^2^) = 0.100
*S* = 1.05
2608 reflections
191 parameters
4 restraints
H atoms treated by a mixture of independent and constrained refinement
Δρ_max_ = 0.16 e Å^−3^
Δρ_min_ = −0.22 e Å^−3^

### 

Data collection: *CrysAlis PRO* (Agilent, 2010 ▶); cell refinement: *CrysAlis PRO*; data reduction: *CrysAlis PRO*; program(s) used to solve structure: *SHELXS97* (Sheldrick, 2008 ▶); program(s) used to refine structure: *SHELXL97* (Sheldrick, 2008 ▶); molecular graphics: *OLEX2* (Dolomanov *et al.*, 2009 ▶); software used to prepare material for publication: *OLEX2*.

## Supplementary Material

Crystal structure: contains datablock(s) global, I. DOI: 10.1107/S1600536812014328/cv5269sup1.cif

Structure factors: contains datablock(s) I. DOI: 10.1107/S1600536812014328/cv5269Isup2.hkl

Supplementary material file. DOI: 10.1107/S1600536812014328/cv5269Isup3.cml

Additional supplementary materials:  crystallographic information; 3D view; checkCIF report

## Figures and Tables

**Table 1:** Hydrogen-bond geometry (Å, °)

*D*—H⋯*A*	*D*—H	H⋯*A*	*D*⋯*A*	*D*—H⋯*A*
N2—H2*A*⋯O3^i^	0.88 (1)	2.25 (1)	3.0186 (18)	146 (2)
N2—H2*B*⋯O1^i^	0.88 (1)	2.00 (1)	2.8427 (18)	159 (2)

Symmetry code: (i) 
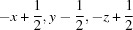
.

**Fig. 1.**
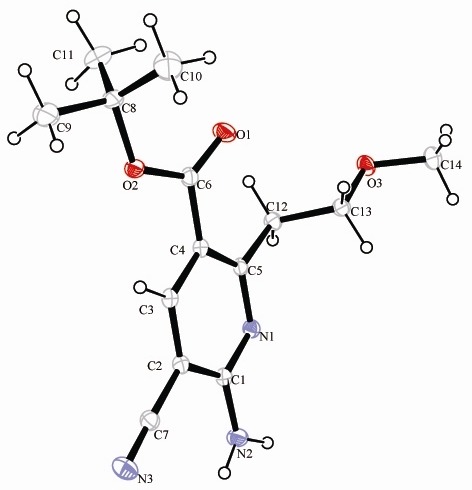
The molecular structure of the title compound. Displacement ellipsoids are drawn at the 50% probability level.

## supplementary crystallographic information

### Comment

Pyridines can be found in many natural products and biologically active compounds (Spurr, 1995). Thus, the synthesis of highly substituted pyridines has attracted much attention (Chun *et al.* 2009, 2011). We synthesized the title compound (I). Herein we present its crystal structure.

In (I) (Fig. 1), all bond lengths and angles are normal and comparable with those observed in the related compound methyl 6-amino-5-cyano-4-(4-fluorophenyl)-2-methylpyridine-3-carboxylate (Wang *et al.*, 2007).

In the crystal structure of (I), intermolecular N—H···O hydrogen bonds (Table 1) link the molecules into chains propagated along the *b* axis.

### Experimental

A mixture of zinc powder (0.65 g) and 3-methoxypropionitrile (0.85 g) in tetrahydrofuran (10 ml) was refluxed, then *tert*-Butyl bromoacetate (1.95 g) was added dropwise. Keep stirring under reflux for 1 h. Ethoxymethylenemalononitrile (1.22 g) was added, the reaction mixture was stirred under reflux for 2 h to afford the title compound (I) (Chun *et al.* 2009, 2011). Single crystals were grown by slow evaporation of a solution of Pet: EtOAc=5:1 at room temperature.

### Refinement

C-bound H atoms were positioned geometrically (C—H 0.95–0.99 Å), and were refined using a riding model, with *U*_iso_(H) = 1.2–1.5 *U*_eq_ (C). N-bound H atoms were located in a difference map and refined freely with *U*_iso_(H) = 1.2 *U*_eq_(N).

### Crystal data

**Table:**

C_14_H_19_N_3_O_3_	*D*_x_ = 1.244 Mg m^−^^3^
*M**_r_* = 277.32	Melting point: 404.16 K
Monoclinic, *C*2/*c*	Mo *K*α radiation, λ = 0.7107 Å
*a* = 10.1155 (4) Å	Cell parameters from 2258 reflections
*b* = 15.2482 (5) Å	θ = 2.9–29.1°
*c* = 19.4882 (6) Å	µ = 0.09 mm^−^^1^
β = 99.853 (3)°	*T* = 130 K
*V* = 2961.59 (18) Å^3^	Block, colourless
*Z* = 8	0.35 × 0.30 × 0.30 mm
*F*(000) = 1184	

### Data collection

**Table:**

Agilent Xcalibur Eos diffractometer	2608 independent reflections
Radiation source: Enhance (Mo) X-ray Source	2094 reflections with *I* > 2σ(*I*)
Graphite monochromator	*R*_int_ = 0.020
Detector resolution: 16.0874 pixels mm^-1^	θ_max_ = 25.0°, θ_min_ = 2.9°
ω scans	*h* = −12→11
Absorption correction: multi-scan (*CrysAlis PRO*; Agilent, 2010)	*k* = −18→10
*T*_min_ = 0.902, *T*_max_ = 1.000	*l* = −15→23
5419 measured reflections	

### Refinement

**Table:**

Refinement on *F*^2^	Primary atom site location: structure-invariant direct methods
Least-squares matrix: full	Secondary atom site location: difference Fourier map
*R*[*F*^2^ > 2σ(*F*^2^)] = 0.040	Hydrogen site location: inferred from neighbouring sites
*wR*(*F*^2^) = 0.100	H atoms treated by a mixture of independent and constrained refinement
*S* = 1.05	*w* = 1/[σ^2^(*F*_o_^2^) + (0.0438*P*)^2^ + 1.1844*P*] where *P* = (*F*_o_^2^ + 2*F*_c_^2^)/3
2608 reflections	(Δ/σ)_max_ < 0.001
191 parameters	Δρ_max_ = 0.16 e Å^−^^3^
4 restraints	Δρ_min_ = −0.22 e Å^−^^3^

### Special details

**Table:**

Geometry. All e.s.d.'s (except the e.s.d. in the dihedral angle between two l.s. planes) are estimated using the full covariance matrix. The cell e.s.d.'s are taken into account individually in the estimation of e.s.d.'s in distances, angles and torsion angles; correlations between e.s.d.'s in cell parameters are only used when they are defined by crystal symmetry. An approximate (isotropic) treatment of cell e.s.d.'s is used for estimating e.s.d.'s involving l.s. planes.
Refinement. Refinement of *F*^2^ against ALL reflections. The weighted *R*-factor *wR* and goodness of fit *S* are based on *F*^2^, conventional *R*-factors *R* are based on *F*, with *F* set to zero for negative *F*^2^. The threshold expression of *F*^2^ > σ(*F*^2^) is used only for calculating *R*-factors(gt) *etc*. and is not relevant to the choice of reflections for refinement. *R*-factors based on *F*^2^ are statistically about twice as large as those based on *F*, and *R*- factors based on ALL data will be even larger.

### Fractional atomic coordinates and isotropic or equivalent isotropic displacement parameters (Å^2^)

**Table:**

	*x*	*y*	*z*	*U*_iso_*/*U*_eq_	
O1	0.21160 (13)	0.04640 (8)	0.29380 (6)	0.0402 (3)	
O2	0.12016 (10)	0.02438 (7)	0.38946 (5)	0.0280 (3)	
O3	0.33430 (10)	−0.04292 (7)	0.12374 (6)	0.0296 (3)	
N1	0.17189 (12)	−0.23015 (8)	0.25819 (6)	0.0241 (3)	
N2	0.16078 (15)	−0.36625 (9)	0.30598 (8)	0.0322 (4)	
H2A	0.1613 (17)	−0.4024 (9)	0.3412 (8)	0.039*	
H2B	0.1820 (17)	−0.3884 (10)	0.2674 (7)	0.039*	
N3	0.11844 (14)	−0.34153 (10)	0.48130 (7)	0.0367 (4)	
C1	0.15937 (14)	−0.27913 (10)	0.31410 (8)	0.0237 (4)	
C2	0.14474 (14)	−0.24020 (10)	0.37841 (8)	0.0233 (4)	
C3	0.14728 (14)	−0.15014 (10)	0.38336 (8)	0.0230 (4)	
H3	0.1386	−0.1227	0.4261	0.028*	
C4	0.16250 (13)	−0.09908 (10)	0.32599 (8)	0.0218 (3)	
C5	0.17249 (13)	−0.14259 (10)	0.26322 (8)	0.0219 (3)	
C6	0.16804 (14)	−0.00242 (11)	0.33331 (8)	0.0250 (4)	
C7	0.13015 (15)	−0.29497 (11)	0.43634 (8)	0.0263 (4)	
C8	0.11812 (17)	0.11866 (10)	0.40839 (9)	0.0303 (4)	
C9	0.0559 (2)	0.11529 (13)	0.47372 (11)	0.0529 (6)	
H9A	0.0480	0.1749	0.4913	0.079*	
H9B	0.1128	0.0802	0.5092	0.079*	
H9C	−0.0334	0.0886	0.4629	0.079*	
C10	0.25917 (19)	0.15437 (14)	0.42359 (11)	0.0505 (5)	
H10A	0.2974	0.1550	0.3806	0.076*	
H10B	0.3143	0.1171	0.4583	0.076*	
H10C	0.2575	0.2142	0.4417	0.076*	
C11	0.03036 (19)	0.16861 (12)	0.35095 (10)	0.0459 (5)	
H11A	0.0773	0.1751	0.3112	0.069*	
H11B	0.0104	0.2268	0.3680	0.069*	
H11C	−0.0536	0.1364	0.3363	0.069*	
C12	0.18509 (14)	−0.09710 (11)	0.19592 (8)	0.0247 (4)	
H12A	0.1426	−0.0385	0.1950	0.030*	
H12B	0.1367	−0.1315	0.1563	0.030*	
C13	0.33020 (14)	−0.08676 (11)	0.18746 (8)	0.0252 (4)	
H13A	0.3796	−0.0524	0.2268	0.030*	
H13B	0.3732	−0.1451	0.1873	0.030*	
C14	0.46425 (17)	−0.04423 (13)	0.10532 (10)	0.0417 (5)	
H14A	0.4621	−0.0133	0.0611	0.063*	
H14B	0.4923	−0.1051	0.1004	0.063*	
H14C	0.5280	−0.0152	0.1418	0.063*	

### Atomic displacement parameters (Å^2^)

**Table:**

	*U*^11^	*U*^22^	*U*^33^	*U*^12^	*U*^13^	*U*^23^
O1	0.0612 (8)	0.0263 (7)	0.0407 (7)	−0.0069 (6)	0.0305 (6)	−0.0014 (6)
O2	0.0394 (6)	0.0213 (6)	0.0262 (6)	−0.0012 (5)	0.0138 (5)	−0.0030 (5)
O3	0.0326 (6)	0.0331 (7)	0.0258 (6)	−0.0025 (5)	0.0128 (4)	0.0028 (5)
N1	0.0260 (7)	0.0240 (7)	0.0236 (7)	−0.0005 (6)	0.0082 (5)	−0.0018 (6)
N2	0.0477 (9)	0.0228 (8)	0.0300 (8)	0.0015 (7)	0.0177 (7)	−0.0008 (7)
N3	0.0444 (9)	0.0367 (9)	0.0296 (8)	−0.0065 (7)	0.0077 (6)	0.0051 (7)
C1	0.0204 (8)	0.0241 (8)	0.0274 (8)	0.0001 (6)	0.0065 (6)	−0.0001 (7)
C2	0.0225 (8)	0.0249 (8)	0.0235 (8)	0.0001 (6)	0.0066 (6)	0.0007 (7)
C3	0.0207 (8)	0.0274 (8)	0.0216 (8)	0.0005 (6)	0.0060 (6)	−0.0013 (7)
C4	0.0185 (7)	0.0245 (8)	0.0231 (8)	0.0004 (6)	0.0059 (6)	−0.0003 (7)
C5	0.0164 (7)	0.0253 (8)	0.0249 (8)	−0.0001 (6)	0.0062 (6)	0.0010 (7)
C6	0.0241 (8)	0.0274 (9)	0.0248 (8)	−0.0003 (7)	0.0078 (6)	−0.0009 (7)
C7	0.0282 (9)	0.0258 (9)	0.0259 (9)	−0.0014 (7)	0.0071 (7)	−0.0029 (8)
C8	0.0399 (9)	0.0211 (8)	0.0324 (9)	−0.0023 (7)	0.0132 (7)	−0.0071 (8)
C9	0.0851 (15)	0.0328 (11)	0.0507 (13)	−0.0069 (10)	0.0392 (11)	−0.0133 (10)
C10	0.0487 (12)	0.0491 (12)	0.0533 (13)	−0.0149 (10)	0.0076 (9)	−0.0227 (11)
C11	0.0542 (12)	0.0304 (10)	0.0526 (12)	0.0095 (9)	0.0080 (9)	−0.0036 (10)
C12	0.0259 (8)	0.0268 (9)	0.0219 (8)	−0.0008 (7)	0.0052 (6)	−0.0002 (7)
C13	0.0304 (9)	0.0246 (8)	0.0223 (8)	0.0020 (7)	0.0094 (6)	0.0022 (7)
C14	0.0431 (11)	0.0426 (11)	0.0470 (11)	0.0023 (9)	0.0290 (8)	0.0050 (10)

### Geometric parameters (Å, º)

**Table:**

O1—C6	1.2072 (18)	C8—C10	1.508 (2)
O2—C6	1.3342 (17)	C8—C11	1.510 (2)
O2—C8	1.4852 (19)	C9—H9A	0.9800
O3—C13	1.4170 (18)	C9—H9B	0.9800
O3—C14	1.4209 (18)	C9—H9C	0.9800
N1—C1	1.3447 (19)	C10—H10A	0.9800
N1—C5	1.339 (2)	C10—H10B	0.9800
N2—H2A	0.880 (12)	C10—H10C	0.9800
N2—H2B	0.883 (12)	C11—H11A	0.9800
N2—C1	1.338 (2)	C11—H11B	0.9800
N3—C7	1.149 (2)	C11—H11C	0.9800
C1—C2	1.417 (2)	C12—H12A	0.9900
C2—C3	1.377 (2)	C12—H12B	0.9900
C2—C7	1.432 (2)	C12—C13	1.513 (2)
C3—H3	0.9500	C13—H13A	0.9900
C3—C4	1.392 (2)	C13—H13B	0.9900
C4—C5	1.410 (2)	C14—H14A	0.9800
C4—C6	1.481 (2)	C14—H14B	0.9800
C5—C12	1.508 (2)	C14—H14C	0.9800
C8—C9	1.515 (2)		
			
C6—O2—C8	121.51 (12)	H9A—C9—H9B	109.5
C13—O3—C14	112.44 (12)	H9A—C9—H9C	109.5
C5—N1—C1	119.64 (13)	H9B—C9—H9C	109.5
H2A—N2—H2B	117.1 (16)	C8—C10—H10A	109.5
C1—N2—H2A	121.9 (11)	C8—C10—H10B	109.5
C1—N2—H2B	119.3 (11)	C8—C10—H10C	109.5
N1—C1—C2	121.50 (14)	H10A—C10—H10B	109.5
N2—C1—N1	116.81 (14)	H10A—C10—H10C	109.5
N2—C1—C2	121.69 (15)	H10B—C10—H10C	109.5
C1—C2—C7	119.56 (14)	C8—C11—H11A	109.5
C3—C2—C1	118.44 (14)	C8—C11—H11B	109.5
C3—C2—C7	121.99 (14)	C8—C11—H11C	109.5
C2—C3—H3	119.8	H11A—C11—H11B	109.5
C2—C3—C4	120.32 (14)	H11A—C11—H11C	109.5
C4—C3—H3	119.8	H11B—C11—H11C	109.5
C3—C4—C5	117.88 (14)	C5—C12—H12A	109.3
C3—C4—C6	119.12 (13)	C5—C12—H12B	109.3
C5—C4—C6	123.00 (14)	C5—C12—C13	111.75 (12)
N1—C5—C4	122.18 (14)	H12A—C12—H12B	107.9
N1—C5—C12	113.27 (13)	C13—C12—H12A	109.3
C4—C5—C12	124.55 (14)	C13—C12—H12B	109.3
O1—C6—O2	123.89 (15)	O3—C13—C12	108.60 (12)
O1—C6—C4	124.35 (14)	O3—C13—H13A	110.0
O2—C6—C4	111.76 (13)	O3—C13—H13B	110.0
N3—C7—C2	177.52 (17)	C12—C13—H13A	110.0
O2—C8—C9	101.62 (13)	C12—C13—H13B	110.0
O2—C8—C10	110.20 (14)	H13A—C13—H13B	108.4
O2—C8—C11	109.60 (13)	O3—C14—H14A	109.5
C10—C8—C9	111.25 (16)	O3—C14—H14B	109.5
C10—C8—C11	112.31 (16)	O3—C14—H14C	109.5
C11—C8—C9	111.34 (16)	H14A—C14—H14B	109.5
C8—C9—H9A	109.5	H14A—C14—H14C	109.5
C8—C9—H9B	109.5	H14B—C14—H14C	109.5
C8—C9—H9C	109.5		
			
N1—C1—C2—C3	−1.8 (2)	C4—C5—C12—C13	93.76 (17)
N1—C1—C2—C7	179.27 (13)	C5—N1—C1—N2	−179.37 (13)
N1—C5—C12—C13	−86.09 (16)	C5—N1—C1—C2	0.9 (2)
N2—C1—C2—C3	178.53 (14)	C5—C4—C6—O1	−18.0 (2)
N2—C1—C2—C7	−0.4 (2)	C5—C4—C6—O2	162.63 (13)
C1—N1—C5—C4	1.0 (2)	C5—C12—C13—O3	−179.41 (12)
C1—N1—C5—C12	−179.11 (12)	C6—O2—C8—C9	179.74 (14)
C1—C2—C3—C4	0.7 (2)	C6—O2—C8—C10	−62.23 (19)
C1—C2—C7—N3	−3 (4)	C6—O2—C8—C11	61.86 (18)
C2—C3—C4—C5	1.2 (2)	C6—C4—C5—N1	177.67 (13)
C2—C3—C4—C6	−178.59 (13)	C6—C4—C5—C12	−2.2 (2)
C3—C2—C7—N3	178 (100)	C7—C2—C3—C4	179.59 (13)
C3—C4—C5—N1	−2.1 (2)	C8—O2—C6—O1	−0.6 (2)
C3—C4—C5—C12	178.08 (12)	C8—O2—C6—C4	178.73 (12)
C3—C4—C6—O1	161.74 (15)	C14—O3—C13—C12	−169.39 (13)
C3—C4—C6—O2	−17.62 (19)		

### Hydrogen-bond geometry (Å, º)

**Table:**

*D*—H···*A*	*D*—H	H···*A*	*D*···*A*	*D*—H···*A*
N2—H2*A*···O3^i^	0.88 (1)	2.25 (1)	3.0186 (18)	146 (2)
N2—H2*B*···O1^i^	0.88 (1)	2.00 (1)	2.8427 (18)	159 (2)

Symmetry code: (i) −*x*+1/2, *y*−1/2, −*z*+1/2.
